# On Sesquichloride of Iron as a Prophylactic of Acute Rheumatism

**Published:** 1871-11

**Authors:** 


### On Sesquichloride of Iron as a Prophylactic of Acute Rheumatism.

By Dr. Anstie.

A considerable space of time has now elapsed since the announcement, by Dr. Russell Reynolds, of his observations on the successful treatment of acute rheumatism by large and frequent doses of the tincture of sesquichloride of Iron. I do not know to what extent this plan of treatment has become generalized; but there have been a good many reports in the medical journals of its employment in different hospitals; and the balance of evidence derivable from these seems distinctly favorable to the method. My own experience of it in fully declared acute rheumatism has not been large. I have treated six cases altogether with the sesquichloride, and in four of these I think the results distinctly bore out the main assertions of Dr. Reynolds, as to the prompt relief of the pains, the limitation of the extent of the mischief, and the shortening of the illness; in the other two, the medicine seemed to have no special effect. But it is not of the use of the sesquichloride in the fully developed acute rheumatism that I now wish to speak. My opportunities of seeing disease on a large scale being chiefly those afforded by the out-patient room, it is rather the first advancings and threatenings of acute rheumatism, than the declared disease, that I am in the habit of seeing. A considerable number of persons present themselves in my out-patient room, during the course of twelve months, suffering from the preliminaries of acute rheumatism; it is one of the small group of really serious diseases (amongst a much larger variety of trivial complaints), which occupy one's attention in out-patient practice, and was formerly a matter of great dissatisfaction to me, from the apparently almost total failure of remedies to produce any effect. Whereas threatenings of gout could be very commonly dealt with in such a manner as to prevent the attack, or render it trivial, the onset of acute rheumatism seemed never to be averted by drugs when once the prodromata had reached the stage which pretty frequently presented itself before me, viz., a more or less obscure aching of several joints,* a yellow sallowness of face, with patches or streaks of dusky redness, blanket-like furring of tongue, an oily moisture of of skin, a distinct though slight elevation both of pulse and temperature, and a certain anxiety of respiration. So far as the history of such patients could be traced, they were almost invariably found to have developed the full symptoms of the acute disease, and very often (after once seeing them in the out-patient room), one encountered them, a few days later, in a ward of the hospital.

Very different have been the results of treatment since I adopted the use of full doses of sesquichloride of iron from the first moment of such cases presenting themselves. During the past twelve months I have done this fully. Whenever a patient has presented himself with articular pain and slight fever that were plainly of the rheumatic and not of the gouty type, he has been at once placed on thirty or forty minims doses of the tincture of sesquichloride, from three to six of which, according to the severity of the symptoms, have been given in each twenty-four hours. I have several times called the attention of the students to the fact, that (unlike what used to happen), these cases now reappear in my out-patient room on my next hospital day; and in the great majority of instances declare themselves greatly relieved. Since July, 1870, I have treated twenty-nine such patients, of whom thirteen had previously had one or more regular attacks of rheumatic fever, for the symptoms now referred to, with full doses of iron; and of these, seventeen have lost all pyrexia and spontaneous joint-pain, within the three or four days elapsing before my next day at the hospital. Only three have, under my own eyes, developed the full acute disease, and been sent to the ward. Of the remaining nine, four disappeared altogether from my knowledge, so that I cannot say what became of them; the other five, though their symptoms were checked, remained in a state of what might be described as subacute rheumatism, during from ten to twenty days.

I cannot help remarking with emphasis on the contradiction to old ideas which is involved in the effect of this iron treatment upon the furred tongue. Of course it becomes speedily blackened; but so far from the furring increasing, or the dryness and foul taste becoming more pronounced, what commonly happens is, that after a few days the epithelial coating falls off in considerable patches, and the tongue soon cleans altogether. I believe the prophylactic treatment of rheumatism by the sesquichloride to be one of our most valuable recent improvements in medicine.—*Am. Practitioner.*—*Medical Archives.*

———:o:———

### On the Physiological action of Bromide of Potassium.

In the *Archives fur Heilkunde*, Heft 2, 1871, is an elaborate paper upon this subject by *G. I. Schouten*, an extended abstract of

* I have, on the contrary, known pain in or near a *single* joint (sometimes simulating neuralgia), with slight fever, sallow skin, etc., yield to iodide and bicarbonate of potash.

